# PHD Inhibitor Vadadustat: The First Drug Therapy for Triosephosphate Isomerase Deficiency

**DOI:** 10.3390/genes17101192

**Published:** 2026-09-26

**Authors:** Emmanuel Scalais, Priyal Patel, Sidney Satterfield, Mylène Donge, Raphael Laschiazza, Arthur Sorlin, Presley Roberts, Joseph R. Figura, Ella R. Donahue, Cindy Cui, Maci Chambers, Kaitlin McClure, Grace Coleman, Naoya Kitamura, James J. Galligan, Laura L. Vollmer, Gregg E. Homanics, Eduard J. van Beers, Andreas Vogt, Michael J. Palladino

**Affiliations:** 1 Pediatric Neurology Unit, Department of Pediatrics, Centre Hospitalier de Luxembourg, L-1210 Luxembourg, Luxembourg; 2 Department of Pharmacology & Chemical Biology, University of Pittsburgh School of Medicine, Pittsburgh, PA 15260, USA; prp179@pitt.edu (P.P.); sls378@pitt.edu (S.S.); pmr45@pitt.edu (P.R.); jof149@pitt.edu (J.R.F.); erd141@pitt.edu (E.R.D.); c.cui@pitt.edu (C.C.); mjc221@pitt.edu (M.C.); kam532@pitt.edu (K.M.); gec81@pitt.edu (G.C.); geh2@pitt.edu (G.E.H.); 3 Pittsburgh Institute for Neurodegenerative Diseases, University of Pittsburgh School of Medicine, Pittsburgh, PA 15260, USA; 4 Clinical Biology Unit, Department of Biochemistry, Centre Hospitalier de Luxembourg, L-1210 Luxembourg, Luxembourg; 5 National Center of Genetics, Laboratoire National de Santé, L-3555 Dudelange, Luxembourg; 6 Department of Pharmacology and Toxicology, University of Arizona College of Pharmacy, Tucson, AZ 85721, USA; kitamura1@arizona.edu (N.K.); jgalligan@pharmacy.arizona.edu (J.J.G.); 7 Department of Computational & Systems Biology, Organ Pathobiology and Therapeutics Institute, University of Pittsburgh School of Medicine, Pittsburgh, PA 15260, USA; llv4@pitt.edu (L.L.V.); avogt@pitt.edu (A.V.); 8 Department of Anesthesiology and Perioperative Medicine, University of Pittsburgh School of Medicine, Pittsburgh, PA 15261, USA; 9 Department of Haematology, Center for Benign Haematology, Thrombosis and Haemostasis, University Medical Centre, 3511 ZK Utrecht, The Netherlands

**Keywords:** TPI deficiency, glycolysis, metabolism, neurology, rare disease, pediatric disease, neuromuscular dysfunction, vadadustat, PHD inhibitor, HIF activation

## Abstract

**Background/Objectives:** Triosephosphate isomerase deficiency (TPI Df) is an ultra-rare autosomal recessive glycolytic disorder characterized by symptoms of hemolytic anemia, progressive neuromuscular dysfunction, and early mortality. The *TPI1^E105D^* mutation (*TPI1* variant p.Glu105Asp) accounts for most cases, though TPI Df can also arise from other pathogenic variants, including compound heterozygous *TPI1* variants such as *TPI1^R5G/f.s^* (p.(Arg5Gly)/p.(Ile151Serfs*3)). *TPI1^R5G/f.s^* presents with an atypical predominantly neurological phenotype characterized by severe dystonia, dysarthria, basal ganglia involvement, and cognitive impairment, without hemolytic anemia or peripheral neuromuscular disease. Regardless of the causative allele(s), reduced mutant TPI protein stability is the central pathogenic mechanism. We previously hypothesized that pharmacological inhibition of prolyl hydroxylase domain (PHD) enzymes, leading to hypoxia-inducible factor (HIF) stabilization, could increase TPI protein levels and attenuate disease severity. **Methods:** We evaluated PHD inhibitors, including vadadustat and roxadustat, in patient-derived *TPI1^E105D/E105D^* fibroblasts and *Tpi1^E105D/E105D^ mouse* models. In addition, vadadustat was administered off-label as compassionate therapy to a TPI Df patient. Studies assessed TPI protein levels, enzymatic activity, hematological parameters, and clinical outcomes. **Results:** In FB303 patient fibroblasts, vadadustat (100 μM) and roxadustat (100 μM) increased TPI protein levels by ~45% and ~68%, and TPI enzymatic activity by ~191% and ~258%, respectively. In *Tpi1^E105D/E105D^* mice, vadadustat significantly increased TPI levels in liver, kidney, and muscle tissues along with partial improvement in neuromuscular function, as assessed by rotarod performance. Supported by these findings, compassionate off-label administration with vadadustat/Vafseo was initiated in a single *TPI1^R5G/f.s.^* patient. Treatment was associated with a progressive increase in residual TPI enzymatic activity, clinical stabilization, and meaningful neurological improvement, predominantly affecting left-sided motor function, including improved upper-limb dexterity, recovery of functional grasping, and complete resolution of myoclonic-like movements. **Conclusions:** This report provides the first *in vivo* evidence that pharmacological stabilization of mutant TPI1 protein may represent a novel therapeutic strategy for TPI deficiency. Furthermore, the findings establish PHD inhibitors, such as vadadustat, as excellent candidates for disease-modifying therapy of TPI Df warranting further development and clinical investigation.

## 1. Introduction

Triosephosphate isomerase deficiency (TPI Df) is an ultra-rare autosomal recessive disorder resulting from mutations in the *TPI1* gene. The disease presents in early childhood with hemolytic anemia, progressive neuromuscular dysfunction, recurrent infections, respiratory failure, and premature death [1,2,3,4,5,6,7,8,9]. Although various pathogenic *TPI1* variants have been identified, the p.Glu105Asp substitution (E105D) is the most common, accounting for around 80% of all diagnosed cases [10,11,12,13]. Not all TPI Df patients follow the same clinical course, and genotype is the key variable. Patients homozygous for *TPI1^E105D^* appear unaffected at birth; however, with time, developmental delays result in missed milestones, and patients experience frequent infections and a gradual deterioration of neurological and muscular function [4]. When *TPI1^E105D^* is inherited together with a null allele, the phenotype is more severe; symptoms are typically present at birth, and neonatal death is common [9]. Regardless of genotype, the molecular defect is the same; disease-associated mutations destabilize the TPI protein while preserving enzymatic function (or at least most of it), and it is the reduction in TPI protein that drives disease progression [14,15,16,17,18]. 

Triosephosphate isomerase (TPI) is a glycolytic enzyme, catalyzing the interconversion of glyceraldehyde-3-phosphate (G3P) and dihydroxyacetone phosphate (DHAP), essential for maximal energy production from glucose [8]. In TPI Df, the loss of functional enzyme disrupts this metabolic balance across all tissues. DHAP cannot be efficiently channeled into downstream metabolism and instead accumulates across tissues, resulting in the formation of methylglyoxal (MGO), a highly reactive toxic di-carbonyl molecule that damages cellular proteins and modifies TPI itself, worsening its instability [19]. As TPI is destabilized, DHAP and MGO accumulate, progressively depleting functional enzyme and likely driving the rapid decline characteristic of TPI Df. 

To model TPI Df *in vivo*, CRISPR-Cas9 was used to create mouse models that recapitulate key aspects of TPI Df pathogenesis [15]. Compound heterozygous *TPI1^E105D/null^* mice carry the most prevalent human pathogenic mutation alongside a frameshift null allele, displaying a markedly shortened lifespan, severe neuromuscular dysfunction, and hemolytic anemia. These mice have ~95% reduced TPI protein levels relative to wild-type littermates, directly reflecting the protein instability that underlies TPI Df pathogenesis [15]. Homozygous *TPI1^E105D/E105D^* mice are viable, exhibit an ~85% reduction in TPI levels that results in neuromuscular phenotypes with slower disease progression and reduced longevity [15,20]. The TPI^E105D^ mutant protein in mice retains significant activity, which is consistent with what has been observed in mutant proteins in numerous human samples. As a result, significant effort has focused on elevating mutant TPI levels as a potential therapeutic strategy for TPI Df [14,15,16,17,18,20,21,22].

TPI deficiency patient-derived fibroblast cells were utilized to complement genetically engineered mouse models. Cells from *TPI1^E105D^* patients exhibit reduced TPI protein levels mirroring the *in vivo* models and assist in evaluating therapeutic interventions of compounds elevating TPI before progression to animal studies. High-throughput compound screening has identified prolyl hydroxylase domain (PHD) inhibitors as compounds capable of increasing TPI protein levels [14,17,18,21], preventing the degradation of hypoxia-inducible factor-α (HIF-α) subunits, and enabling downstream effects that upregulate glycolytic enzymes, enhance erythropoiesis, and promote cellular stress responses. PHD inhibitors such as vadadustat (AKB-6548 [i.e., vadadustat; Akebia Therapeutics, Cambridge, MA, USA]) and roxadustat (FG4592 [i.e., Roxadustat; Kyntra Bio, San Francisco, CA, USA]) emerged as particularly promising candidates, as they are orally bioavailable and are currently being used to treat anemia in chronic kidney disease (CKD) patients, offering an established safety profile. We reasoned that these PHD inhibitors might provide therapeutic benefit in TPI Df by increasing mutant TPI protein levels through HIF-mediated activation of *TPI1* expression [21].

To date, therapeutic options for TPI Df remain purely supportive, and no disease-modifying treatment has been reported in human patients. We investigated the effect of PHD inhibitors (vadadustat and roxadustat) on TPI^E105D^ protein levels, using previously established human *TPI^E105D^*^/E105D^ cells (FB303), and genetically engineered *TPI^E105D/E105D^* mice. Treatment with either compound significantly increased expression of the TPI mutant protein and enzymatic activity levels. Together with our previous findings demonstrating that PHD inhibitors increased levels of mutant TPI in *TPI1^R5G/f.s^* cells [21], these data provided the rationale for a compassionate use of vadadustat (trade name Vafseo) in a single patient with TPI deficiency associated with compound heterozygous *TPI1* variants. The data reported provide the first *in vivo* evidence supporting pharmacological rescue of TPI Df. These findings demonstrate that PHD inhibitors are promising candidates for disease-modifying therapy and provide a strong rationale for further development and clinical investigation.

## 2. Materials and Methods

### 2.1. Cell Culture

Patient fibroblasts named FB303 were previously published [14,18]. FB303s were confirmed to be homozygous for the “common” mutation, *TPI1^E105D^*. FB902 (*TPI1^WT^/TPI^WT^*), FB826 (*TPI1^WT^/TPI^WT^*), FB755 cells (Q181P/E105D), and FB753 (*TPI1^Q181P^/TPI^WT^*) were also previously published (PMID: 35315486). Standard cell culture incubation procedures (37 °C, 5% CO_2_) were followed to culture these fibroblasts in complete media (20% fetal bovine serum (FBS, Thermo Fisher Scientific, Waltham, MA, USA) Dulbecco’s Modified Eagle Medium (DMEM, Thermo Fisher Scientific, Waltham, MA, USA), 2 mM L-Glutamine (Gibco/Thermo Fisher Scientific, Waltham, MA, USA), 100 U penicillin/100 μg streptomycin/mL (Sigma-Aldrich, Burlington, MA, USA), and non-essential amino acids (HyClone, Logan, UT, USA). 

### 2.2. Cellular Pharmacology and Cell Counting

Cell counting methods were previously described [17,21]. In short, cells were counted on a hemocytometer and plated at a density of 200,000 cells per 100 mm dish. Molidustat (Selleckchem, Houston, TX, USA), daprodustat (Selleckchem, Houston, TX, USA), roxadustat (AK Scientific, Union City, CA, USA), and vadadustat (Akebia Therapeutics, Cambridge, MA, USA) were used. After 24 h, media was aspirated from plates, and 10 mL of new media containing the test drug dissolved in a 1% DMSO solution, or 1% DMSO vehicle control (Sigma-Aldrich, Burlington, MA, USA), was added to each plate, with an “*n*” of 8 for each condition. After a 48 h incubation period, cells were harvested and lysed following previously reported methods [17,21].

### 2.3. Indirect Immunofluorescence

Immunostaining and high-content imaging were performed on *TPI1^E105D/E105D^* and *TPI1^Q181P/E105D^* patient fibroblasts exactly as described [18].

### 2.4. Western Blot

*In vitro studies:* Western blots were performed as previously reported [21]. 

*In vivo studies:* Flash-frozen mouse tissues were lysed in 800 μL of RIPA buffer (Thermo Scientific, Waltham, MA, USA) via an IKA Ultra-Turrax T8 mechanical homogenizer (IKA, Staufen, Germany). Samples were subsequently centrifuged for 20 min at 12,000× *g*, and the supernatant was collected. A BCA assay (Thermo Scientific, Waltham, MA, USA) was utilized to determine protein concentrations of the lysates. Samples were loaded onto a 12% acrylamide gel with 15 μg of total protein per well, then run for 50 min at 200 V in 1× SDS-PAGE running buffer (Fisher BioReagents, Pittsburgh, PA, USA). Proteins were transferred onto a PVDF membrane (Thermo Scientific, Waltham, MA, USA) using a semi-wet transfer tank (BioRad, Hercules, CA, USA) at 100 V for 1 h at 4 °C. After transfer, blots were blocked using LiCor Intercept Protein-Free Blocking Buffer (LiCor Bioscience, Lincoln, NE, USA) for 1 h. Primary antibodies for rabbit anti-TPI (1:5000, Santa Cruz, Dallas, TX, USA), mouse anti-β-actin (1:1000, Abcam, Waltham, MA, USA), or mouse anti-GAPDH (1:1000, Invitrogen, Waltham, MA, USA) were added to the blot via blocking buffer and incubated overnight at 4 °C. The following day, blots were washed 5 times for 5 min each in PBS-T (1× PBS with 0.05% Tween 20). Secondary antibodies for goat anti-mouse 680 (Invitrogen, Waltham, MA, USA, 1:20,000) and donkey anti-rabbit 800 (LiCor Bioscience, Lincoln, NE, USA, 1:20,000) were added to LiCor Intercept Protein-Free Blocking Buffer with 0.1% Tween 20. Blots were incubated in the dark with secondary antibodies for 1 h at room temperature. Blots were then washed again 5 times for 5 min each in PBS-T. Afterward, blots were imaged via the LiCor Odyssey cLx, and Image Studio version 5.2 (LiCor) was used to analyze Western blot results.

### 2.5. TPI Enzymatic Activity Assay

Fibroblasts were plated in 10 cm dishes with 200,000 cells per plate. After a 24 h adherence period, old media was aspirated, and FB303s were treated with either 100 μM roxadustat or vadadustat, as well as a 1% DMSO vehicle control diluted in 10% FBS DMEM. After each plate received 10 mL of drug treatment, plates were placed back in the incubator for 48 h. After this incubation period, FB303 cells were harvested following the protocol described in the cell counting and drug treatment section. The TPI activity assay, consisting of a standard curve, TPI positive controls, and diluted samples, was prepared and plated on a microplate following previously reported methods [17]. In brief, diluted samples, standards, and positive controls were treated with a TPI reaction mix consisting of TPI enzyme, TPI substrate, and a developer solution to form a colored product in which absorbance at 450 nm can be measured. Once in the microplate reader (Benchmark Scientific, Inc., Sayreville, NJ, USA), the rate of DHAP to G3P conversion is measured every 3 min for 40 min total. Activity was expressed in U/mg. 

### 2.6. Animal Care and Approval

All animal procedures were approved by the University of Pittsburgh Institutional Animal Care and Use Committee (IACUC) and conducted in compliance with the NIH Guide for the Care and Use of Laboratory Animals. Animal use justification; power calculations; humane treatment; experimental design; housing conditions; health, pain/distress, and endpoint monitoring; and all procedural details were reviewed and approved by the IACUC oversight committee for compliance with ethical standards. No animals were excluded from the study or analysis. Every other animal of the correct genotype was assigned to experimental and negative control groups. For behavioral studies, the technician performing the studies was blinded to the grouping. Mice were housed under pathogen-free conditions in individually ventilated cages on a 12 h light/dark cycle with *ad libitum* food and water. Animals were monitored regularly, and those exhibiting severe neuromuscular phenotypes were provided with moistened food and hydrogels on the cage floor to facilitate access.

### 2.7. Genotyping

The *TPI1^E105D/E105D^* knock-in mouse model was generated by CRISPR-Cas9 gene editing as previously described [15]. Genomic DNA was isolated from ear-snip biopsies at approximately two weeks of age using QuickExtract DNA Extraction Solution (Lucigen, Middleton, WI, USA) and amplified by PCR using GoTaq™ DNA Polymerase (Promega, Madison, WI, USA) with the following primers: forward 5′-GGTGGTTTGTGCACCGCCCACCGC-3′ and reverse 5′-CCTTTGACCTCCCGCCTCCATCCC-3′. Purified PCR products (QIAquick PCR Purification Kit, Qiagen, Hilden, Germany) were sent to Genewiz (Azenta Life Sciences, South Plainfield, NJ, USA) for Sanger sequencing, and genotypes were confirmed using Lasergene software version 18 (DNASTAR, Madison, WI, USA).

### 2.8. Tissue Distribution Study

A tissue distribution study was conducted in C57BL/6J wild-type mice (either sex, aged 50–80 days, weighing 25–30 g). Mice were randomly assigned to three groups: blank (*n* = 3), roxadustat (100 mg/kg; *n* = 24), and vadadustat (175 mg/kg; *n* = 24). Both compounds were formulated in 0.25% hydroxypropyl methylcellulose (HPMC, Thermo Fisher scientific, Waltham, MA, USA) and 0.1% Tween 80 (Thermo Fisher scientific, Waltham, MA, USA) in sterile water and administered orally. Mice were sacrificed at 0.08, 0.25, 0.5, 1, 2, 4, 8, and 24 h post-dose, and tissues and blood were collected for analysis. Plasma samples were extracted twice with 4 volumes of ice-cold MeOH containing 0.1% formic acid and 750 pmol ^13^C_2_^15^N vadadustat (internal standard, Acanthus Research, Mississauga, ON, Canada) or 600 pmol roxadustat d5 (Cayman Chemical, AnnArbor, MI, USA). Samples were sonicated, vortexed, and centrifuged to remove protein and debris. The resulting supernatants were transferred to a new tube, and the combined supernatants were evaporated to dryness using a SpeedVac at room temperature. For tissue samples, 100 mg of tissue was homogenized in 400 µL of 80% MeOH + 0.1% formic acid and internal standard using a bead mill. The internal standards were added at the following amounts: kidney and liver received 750 pmol ^13^C_2_^15^N vadadustat or 600 pmol roxadustat d5; muscle and brain received 300 pmol ^13^C_2_^15^N vadadustat or 300 pmol roxadustat d5. Samples were then sonicated in a water bath and spun down at max speed. The solvent was transferred to a new tube and evaporated to dryness using a SpeedVac at room temperature. The dried metabolite extraction was resuspended in 150 µL of 80:20 ddH_2_O:ACN +0.1% formic acid, and samples were chromatographed using a Shimadzu LC system equipped with a 50 × 2.1 mm, 3 μm particle diameter Atlantis C_18_ column (Waters, Milford, MA, USA) at a flow rate of 0.5 mL/min. Buffer A (0.1% formic acid in H_2_O) was held at 80% for 30 s, then a linear gradient to 100% solvent B (0.1% formic acid in ACN) was applied over 3.5 min. The column was held at 100% B for 3 min and then washed and equilibrated at 80% A for 1.5 min. Multiple reaction monitoring (MRM) was conducted in positive ion mode using an AB SCIEX 6500 QTRAP+ (AB Sciex Marlborough, MA USA) with the transitions in Table 1.

### 2.9. Mouse Tissue Collection

Terminal body weights of animals were recorded prior to dissection. At each time point, animals were euthanized via isoflurane overdose. Dissection was initiated with a T-shaped incision across the abdomen. The diaphragm was excised to expose the thoracic cavity, and a cardiac puncture was performed on the left ventricle with a 1-inch 23-gauge needle (Becton, Dickinson and Company (BD), Franklin Lakes, NJ, USA; BD Cat No: 309588) for blood collection. Whole blood was collected in a clean tube and kept undisturbed at room temperature for 30 min. The clotted blood was centrifuged at 5000 rpm (2000× *g*) for 10 min, and the separated serum was stored at −80 °C. Following blood collection, liver, kidney, skeletal muscle, and brain were isolated, cleaned in normal saline, and 100 mg of tissue was snap-frozen and stored at −80 °C. All the serum and tissue samples were shipped on dry ice to Touchstone Biosciences (Plymouth Meeting, PA, USA) for further analysis.

### 2.10. Murine Drug Administration

*Tpi1^E105D/E105D^* mice (either sex, 30 days of age and weighing 13–14 g) were randomly assigned into two groups (*n* = 8 per group). Starting at age 30, group I mice received a 5 mL/kg dose of DMSO vehicle control (0.25% HPMC and 0.1% Tween 80) administered orally once daily for 20 days, and group II mice received 175 mg/kg vadadustat (formulated in 0.25% HPMC and 0.1% Tween 80) orally once daily for 20 days. On day 21, mice were euthanized by isoflurane overdose. An abdominal incision was performed, and liver, kidney, muscle, brain, and forelimb were collected. Each tissue was rinsed in normal saline, and 100 mg of each tissue was snap-frozen in liquid nitrogen and stored at −80 °C until further analysis.

### 2.11. Mouse Motor Function Tests

Neuromuscular coordination was assessed using the rotarod test at postnatal days 30, 35, 40, 45, and 50. Testing was performed using a Panlab Rotarod (Model LE8205, Holliston, MA, USA) set to an accelerating speed protocol from 5 to 35 rpm over a 180 s period. Each animal underwent a practice session to familiarize them with the apparatus prior to the recorded trials. Three test trials were conducted per animal with a 5 min rest period between each trial. The rotarod was cleaned between trials to prevent any behavioral alterations.

### 2.12. Animal Bloodwork Analyses

Whole blood samples were collected by cardiac puncture of the left ventricle and were stored with EDTA at 4 °C until being shipped to IDEXX BioAnalytics (Westbrook, ME, USA) for analyses as previously reported [15].

### 2.13. Off-Label Usage of Vadadustat

This study details the off-label administration of Vafseo, an authorized hypoxia-inducible factor prolyl hydroxylase inhibitor, for treatment in a patient with TPI deficiency. In addition to approval by the Ministry of Health in Luxembourg (Ministere de la Santé) and the CNS (Caisse Nationale de Santé), this usage is in accordance with EU Directive 2001/83/EC and all relevant national regulations. This prescription was classified as off-label use given the absence of approved disease-modifying therapies for this ultra-rare disorder. Informed consent was obtained from the patient’s legal guardians (in this case, the parents) following a comprehensive disclosure of the off-label nature, potential risks, and anticipated benefits. Additionally, age-appropriate patient assent was secured from the child prior to initiation, respecting their developing autonomy. Clinical monitoring, including hemoglobin assessment and adverse event reporting, was conducted in strict adherence to pharmacovigilance standards applicable to off-label prescribing within the European regulatory framework and reported here as well.

### 2.14. Statistics

All statistical tests were conducted using GraphPad Prism 10 (version 10.6.1). For whole-protein cell lysate Western blot analysis and TPI activity analysis, a Kruskal–Wallis non-parametric statistical test was used to analyze Figure 1B, Figure 2B, and Figure 3B. A two-tailed Mann–Whitney test was used in the analysis of Figure 1D. 

## 3. Results

### 3.1. PHD Inhibitors Cause Dose-Dependent Increases in TPI Protein in FB303 Cells

Previous drug discovery efforts identified PHD inhibitors as promising candidates for the treatment of TPI Df [18]. The in vitro efficacy of several PHD inhibitors was recently tested in patient cells with a newly discovered TPI Df allele, *TPI1^R5G^* [21]. Here, we investigated whether these drugs work in FB303 patient cells homozygous for the most common TPI Df allele *TPI1^E105D^* and in compound heterozygous FB755 patient cells with the genotype *TPI1^Q181P/E105D^.* Immunofluorescence was used to conduct a 10-point dose–response with four different PHD inhibitors: daprodustat, molidustat, roxadustat, and vadadustat (Figure 1). All four drugs produced a dose-dependent increase in TPI levels in both FB303 and FB755 cells (Figure 1A). The percent increase observed and the EC50 are provided (when a plateau was evident) for FB303 (Figure 1B) and FB755 (Figure 1C).

### 3.2. Confirming Vadadustat and Roxadustat Efficacy by Detection of TPI Protein Levels in FB303 Cells

To confirm the effect of PHD inhibitors on patient fibroblasts using an orthogonal, non-fluorescent assay, we conducted Western blot assays with FB303 cells. The EC50 data from immunofluorescence experiments suggested that 100 μM should provide a good approximation of the maximal effect of roxadustat and vadadustat. Treatment with roxadustat and vadadustat at 100 μM resulted in a significant increase in relative TPI protein levels in FB303 cells relative to DMSO vehicle control treatments (Figure 2A and Figure 2C, respectively). Representative blots are shown (Figure 2B,D). Roxadustat produced a ~65% increase, and vadadustat produced an ~45% increase in TPI protein levels, consistent with what was observed in the immunofluorescence experiments (Figure 1B,C). 

### 3.3. PHD Inhibitor Treatment Increases TPI Enzyme Activity in TPI Df FB303 Cells 

Both roxadustat and vadadustat treatment significantly increased relative TPI protein levels, which led to further investigation of how these compounds impact TPI enzyme activity in human patient cells. FB303 cells treated with 100 μM of roxadustat and a 1% DMSO vehicle control for 48 h were harvested and lysed to measure the enzymatic activity of TPI. TPI activity was normalized to total milligrams of protein, and units of TPI activity were expressed in U/mg. 

Treatment with roxadustat increased TPI activity in FB303 cells by 250% (Figure 3A,C). Vadadustat increased TPI protein levels as well, although not to the same magnitude as roxadustat. Thus, we asked if the increase in TPI protein levels with 100 μM vadadustat would similarly lead to a significant change in TPI activity relative to vehicle controls. Treatment with 100 μM vadadustat resulted in a 191% increase in TPI activity (Figure 3B,D).

### 3.4. Therapeutically Relevant Increase After PHD Inhibitor Treatment

The significant increases in TPI protein and activity levels seen after treatment with vadadustat led us to explore whether these increases were therapeutically relevant and would elevate TPI levels to those in human cells from an unaffected heterozygous individual (FB753; *TPI1^Q181P/WT^*) or potentially to those in WT fibroblasts. To assess the extent of the rescue in TPI protein, we conducted Western blot assays that measured TPI levels across 2 different untreated WT cell lines, FB902 and FB826, a heterozygous cell line FB753 (*TPI1^Q181P/WT^*), and untreated and vadadustat-treated FB303 patient cells (*TPI1^E105D/E105D^*). FB303 cells were treated with 100 μM vadadustat, and all other cell lines were treated with a 1% DMSO vehicle control for comparison. Consistent with our previous results (Figure 2), we found that vadadustat caused TPI protein levels to significantly increase in FB303 cells (Figure 4). Importantly, the TPI protein levels in vadadustat-treated cells were not significantly different than WT TPI levels (FB902 or FB826) or the levels observed in FB753 heterozygote cells. These data suggest the increases observed in vitro are therapeutically relevant in magnitude and should be investigated *in vivo*.

### 3.5. Vadadustat In Vivo Efficacy Testing in TPI1^E105D/E105D^ TPI Df Mice

Previous published animal studies were used to guide dosage selection for vadadustat efficacy studies [23,24]. Rotarod performance was measured in *TPI1^E105D/E105D^* mice at days 30, 35, 40, 45, and 50 following treatments with vadadustat (175 mg/kg) or DMSO vehicle control. Across all time points, vadadustat-treated mice consistently showed greater average latency to fall than the DMSO vehicle control (Figure 5). The most pronounced effect was observed at day 40, where vadadustat-treated mice remained on the rotarod significantly longer (~71 s) than the DMSO vehicle control (~41 s) (*p* = 0.037). A trend toward improved performance was also noted at day 35, though this did not reach statistical significance (*p* = 0.057). On days 30, 45, and 50, differences between groups were not statistically significant (*p* = 0.164, *p* = 0.484, and *p* = 0.201, respectively). Taken together, the rotarod data support a modest improvement in neuromuscular function with vadadustat treatment, most evident at day 40.

### 3.6. PHD Inhibitor Treatment Increases TPI Protein Levels in Tpi1^E105D/E105D^ Mice 

TPI protein levels were assessed by Western blot in liver, kidney, muscle, and brain tissue collected from *Tpi1^E105D/E105D^* mice treated daily for 20 days with vadadustat (175 mg/kg) or DMSO vehicle control. The most substantial increases were observed in the liver and kidney, where TPI protein was elevated approximately 4.8-fold and 3.2-fold, respectively, both reaching statistical significance (*p* = 0.0002) (Figure 6). In muscle, TPI protein was also significantly elevated at approximately 1.35-fold compared to DMSO vehicle control (*p* = 0.0104; Figure 6). In contrast, brain TPI protein levels were not significantly different in vadadustat-treated mice relative to DMSO vehicle control (*p* = 0.0878; Figure 6). Collectively, these findings suggest that the pharmacological effect of vadadustat on TPI protein levels is most pronounced in peripheral tissues, and the effect was not detectable in the brain.

Along with increases in TPI protein levels following PHD inhibitor treatment in *TPI1^E105D/E105D^* mice, blood was obtained during tissue collection for hematologic analyses. Consistent with hemolytic anemia symptoms of TPI Df, DMSO vehicle control-treated *Tpi1^E105D/E105D^* mice exhibited baseline hemoglobin of ~10.5 g/dL (Figure 7). *TPI1^E105D/E105D^* mice dosed daily with oral vadadustat (175 mg/kg) for 20 days showed hemoglobin levels trending toward improvement (10.5 vs. 11.5 g/dL). This is similarly seen with hematocrit levels (44% vs. 50%); however, these did not reach statistical significance (*p* > 0.05, *n* = 6 per group). Reticulocyte counts remained stable, indicating stable red blood cell turnover. These findings suggest vadadustat provides limited erythropoietic benefit in homozygous *Tpi1^E105D/E105D^* mice.

### 3.7. Tissue Distribution Analysis of Vadadustat and Roxadustat

As the effect of vadadustat treatment on TPI levels varied significantly between tissues, we investigated the pharmacokinetic profile of both vadadustat and roxadustat. Tissue distribution studies were performed for vadadustat and roxadustat, with serum and several disease-relevant tissues being collected at various time points over a 24 h period (Figure 8). Both PHD inhibitors reached peak serum concentrations within the first hour post-administration (~400–450 nmol/mg) (Figure 8). Vadadustat is broadly distributed amongst peripheral tissues, with the highest concentrations in the liver (~100–150 nmol/mg) and kidney (~100–200 nmol/mg) (Figure 8, blue). Importantly, vadadustat achieved measurable concentrations in skeletal muscle (~20 nmol/mg), a tissue critical for addressing the neuromuscular manifestations of TPI Df (Figure 8D, blue). Although it was detectable in the brain, it reached modest levels (~5–8 nmol/mg; Figure 8E, blue) that may not reflect true brain penetration, as there is a small vascular volume within the tissue sample. Roxadustat exhibited similar tissue distribution patterns with generally higher peak concentrations across all tissues (liver ~350–400, kidney ~400–500, muscle ~30–35, brain ~10 nmol/mg) (Figure 8, red). The pharmacokinetic profile of roxadustat closely resembles that of vadadustat, with both drugs exhibiting clearance by hour 24, supporting once-daily dosing regimens. These findings demonstrate that PHD inhibitors achieve multi-tissue distribution appropriate for a systemic TPI Df therapy but lack significant brain penetration. 

### 3.8. Compassionate Off-Label Use of Vadadustat in a 16 YO Female Pediatric TPI Df Patient

- *Dose escalation and safety of Vafseo (vadadustat) with biologic monitoring:* Previously, a pediatric patient with a novel form of TPI Df lacking anemia but with severe neurological and locomotor impairment was reported [17]. The patient’s condition was progressive and worsened significantly over the past several years, warranting compassionate use and repurposing Vafseo as a potential new therapy for TPI Df [21]. The use in this patient was approved by the Ministry of Health in Luxembourg (Ministere de la Santé) and the CNS (Caisse Nationale de Santé). As this compassionate use was the first known treatment of a pediatric patient as well as the first administration of Vafseo to a TPI Df patient, a slow dose escalation starting with 75 mg/day was administered with comprehensive medical supervision. Routine clinical and biological monitoring occurred throughout the treatment escalation and revealed no hematologic, hepatic, renal, or metabolic toxicity (Table 2). Due to the black box warning on the Vafseo package, Hb was monitored to ensure it never exceeded the upper limit of normal based upon the patient’s age and sex. Importantly, no adverse events occurred during the dose escalation and throughout the treatment. Also, Hgb did not rise significantly throughout the treatment (Figure 9). Prior to treatment, TPI activity assay demonstrated residual erythrocyte enzymatic activity of 42 U/g Hb, and this was monitored several times throughout the dose escalation. TPI activity followed a progressive increase to 103.6 U/g Hb at month 6, reaching a peak value of 237.4 U/g Hb at month 10, with persistent elevation at 171.5 U/g Hb at month 11. Although TPI activity did not remain at the levels observed at 10 months, this assay has a high level of variability, and data from more than one patient will be needed to determine the significance of this observation. Taken together, these findings nonetheless suggest a pharmacodynamic effect of Vafseo associated with partial *in vivo* restoration of residual TPI enzymatic activity.
- *Neurological evolution under treatment:* The patient presented with bilateral but markedly asymmetrical dystonia, predominantly affecting the right upper limb and the left lower limb with contracture. Dystonia worsened during voluntary movements and additionally involved facial musculature, cervical posture, and speech production, resulting in significant dysarthria. Following Vafseo therapy, clinically meaningful neurological improvement was observed, predominantly affecting the left side. Motor examination demonstrated improved dexterity of the left upper limb, with recovery of functional grasping and preserved thumb–index pincer function. Dystonia of the right upper limb was less pronounced, and the previously observed myoclonic-like movements had completely resolved. Residual neurological deficits remained, consisting predominantly of dystonic flexion contracture of the right hand and persistent asymmetric dystonia, more pronounced in the left lower limb. She ambulates with a walker or with adult assistance but remains unable to walk independently. She tolerates 60 min physiotherapy sessions; however, dystonia worsens after approximately 3 h of intensive rehabilitation. Muscle strength remained normal, except for persistent weakness of left ankle dorsiflexion. Deep tendon reflexes were present and symmetrical, with no pyramidal or Babinski signs. Speech remains partially intelligible because of persistent dysarthria, although the patient could formulate sentences and demonstrates emerging reading abilities.

## 4. Discussion

TPI Df is a progressive and fatal childhood disease for which no pharmacological treatment currently exists. Affected children receive dietary modifications and nutritional supplementation as primary therapeutic strategies; however, neither has a demonstrated benefit for survival or quality of life. As the disease advances, supportive interventions, including feeding tubes and ventilatory support, may prolong life but do not address the underlying disease or restore meaningful function. We previously identified HIF-activating compounds as a lead therapeutic class for TPI Df through high-throughput screening in a human cellular model of the disease [18] As a class of HIF-activating compounds, PHD inhibitors stabilize the transcriptional activator HIF-1α by preventing its degradation, thereby upregulating expression of downstream target genes, including *TPI1*. Some PHD inhibitors have already been approved in various countries for CKD-associated anemia, and generally they have a good safety profile, supporting their potential repurposing for TPI Df. The present study evaluates clinically developed PHD inhibitors, including vadadustat, for their ability to stabilize TPI protein levels and activity in patient fibroblasts. Vadadustat was also tested for efficacy in an established mouse model of TPI Df and increased TPI protein levels across several tissues in *TPI1^E105D/E105D^* mice and improved neuromuscular function as assessed by the rotarod. These studies prioritized vadadustat, as this compound is approved for use in Europe and the USA, where many TPI Df patients are known to exist. It should be noted that other PHD inhibitors/HIF activators would be predicted to be effective as a TPI Df treatment, as they work through the same established mechanism. Furthermore, other PHD inhibitors/HIF activators may be more effective if they have better tissue distribution, especially in the neuromuscular system. 

A dose–response analysis in patient-derived fibroblasts validated several PHD inhibitors’ ability to increase TPI protein levels in a concentration-dependent manner for both FB303 and FB755 cells. All compounds tested resulted in TPI increases, with roxadustat exhibiting slightly higher in vitro potency (EC50 ~2.5 µM) compared to vadadustat (EC50 ~9.3 µM). Both EC50s were well within achievable levels reported in the literature for this class of drug. Consistent upregulation of TPI across patient genotypes with PHD inhibitors validates this therapeutic approach and provides a potential framework for clinical dosing. Following dose–response characterization, TPI protein levels and activity enhancement were measured in FB303 patient fibroblasts, which are homozygous for the most common *TPI1* variant. Concentrations of 100 µM were used for roxadustat and vadadustat treatment and resulted in significantly increased TPI protein levels, with corresponding increases in enzymatic activity of approximately 258% and 191%, respectively. The upregulation of TPI protein levels and corresponding increases in enzymatic activity highlight the capability of PHD inhibitors to increase functional enzyme via transcription activation. This addresses a key pathogenic mechanism of TPI Df: mutant protein instability leading to insufficient enzymatic activity. Furthermore, TPI levels in vadadustat-treated cells were compared to those from WT fibroblasts or patient cells from an unaffected heterozygous individual (FB753; Q181P/WT). Western blot results revealed that vadadustat treatment significantly increased TPI protein levels up to TPI levels of two WT cell lines. The achievement of wild-type TPI levels is therapeutically significant as it suggests potential for true disease modification rather than symptomatic management and establishes a clear therapeutic target for clinical dosing strategies.

Neuromuscular dysfunction is a prominent clinical feature of TPI Df, and the rotarod assay was used to evaluate whether vadadustat treatment is capable of causing functional improvement in animals. Vadadustat significantly improved rotarod performance at postnatal day 40 (i.e., following 10 days of treatment) in *Tpi1^E105D/E105D^* mice, where treated animals remained on the apparatus substantially longer than the DMSO vehicle control. A near-significant trend toward better performance was observed at day 45, and treated mice maintained higher average latency values at every time point tested. However, only day 40 was significant, and the result did not persist as the disease progressed. The treatment improved TPI protein levels in several tissues, including kidney, liver, and muscle, but not in the brain. Skeletal muscle is an important contributor to rotarod performance, and an increase in TPI protein in this tissue likely accounts for the motor improvement seen at day 40. The loss of significant benefit at later time points most likely reflects continued disease progression and the absence of TPI elevation in the brain. The lack of an increase in TPI in the brain is likely a result of poor blood–brain barrier penetration of vadadustat, restricting distribution to the periphery. Rotarod performance depends on both peripheral muscle and central motor function, and without raising brain TPI protein levels, peripheral TPI elevation appears unable to compensate for the progressive central brain dysfunction. These findings support vadadustat as a viable but partial therapeutic candidate for TPI Df, and suggest that more complete disease modification will require strategies capable of restoring TPI in both peripheral tissues and the brain.

Vadadustat produced the most substantial TPI protein increases in the liver and kidney, at approximately 4.8-fold and 3.2-fold, respectively. Both organs are highly vascularized and among the most responsive tissues to HIF pathway activation. The magnitude of these fold changes also reflects the substantially reduced TPI baseline in TPI Df, where even moderate transcriptional upregulation produces large relative increases. Restoring TPI in these metabolically active organs reduces the enzymatic deficit that underlies disease pathogenesis. The kidney response carries additional relevance, as PHD inhibition in this organ also activates erythropoietin production, a dual effect that may benefit both the enzymatic and hematological components of TPI Df. Vadadustat treatment in *Tpi1^E105D/E105D^* mice resulted in trends toward improved hemoglobin and hematocrit levels with stable reticulocyte counts indicating no increase in red blood cell destruction. While these improvements did not reach statistical significance in the *Tpi1^E105D/E105D^* mice, they are consistent with the expected erythropoietic benefits of PHD inhibition and support vadadustat’s safety profile in TPI Df models. A dose-finding study and efficacy testing at additional doses may be able to optimize future studies of vadadustat in TPI Df mice. 

Pharmacokinetic analysis of vadadustat and roxadustat revealed measurable concentrations in peripheral tissues relevant to TPI Df. Both compounds exhibited similar tissue distribution patterns with the highest levels in liver and kidney. The high concentration of these PHD inhibitors in the kidney is consistent with the drugs having been developed for their erythropoietic effects, as kidneys are key sites of HIF-responsive erythropoietin production. Furthermore, both drugs achieve moderate levels in skeletal muscle and minimal brain penetration. The particularly low brain tissue concentrations observed for both PHD inhibitors suggest little to no CNS penetration. Although the drugs were detectable within brain samples, this could have been due to residual compound in the vascular compartment; in any case, levels achieved appear insufficient to elicit a therapeutic effect. Nevertheless, the restricted CNS penetration suggests that the benefits observed in *Tpi1^E105D/E105D^* mice with vadadustat treatment likely result from muscle TPI improvements and/or benefits from peripheral HIF activation in various tissue systems. Collectively, these data provide strong proof of concept for developing HIF activators with broad distribution, particularly throughout the neuromuscular system, for the treatment of TPI Df.

Although pharmacokinetic studies in mice suggest limited brain penetration of vadadustat, the mechanism underlying the clinical improvement observed in this patient remains uncertain. The neurological benefit was characterized predominantly by improved motor control, including improved left upper-limb dexterity, recovery of functional grasping, complete resolution of myoclonic-like movements, and partial reduction in dystonia, whereas muscle strength remained essentially unchanged. These findings suggest that the clinical response cannot be explained solely by improved skeletal muscle function and may reflect indirect systemic effects, differences in drug distribution between mice and humans, or other mechanisms that remain to be elucidated.

This patient’s case differs substantially from classical TPI deficiency associated with *TPI1^E105D^*. First, despite severe TPI enzymatic deficiency, no hemolytic anemia or systemic manifestations developed. Second, unlike classical cases characterized by progressive peripheral neuromuscular degeneration, our patient demonstrated a selective central nervous system phenotype dominated by dystonia and basal ganglia involvement. Third, DHAP concentrations remained normal, suggesting genotype-dependent biochemical heterogeneity. These observations support the hypothesis that distinct *TPI1* variants may differentially affect catalytic activity, protein stability, tissue-specific vulnerability, and downstream metabolic consequences. The mechanism underlying Vafseo’s benefit in this patient is not completely understood; however, we have shown in TPI Df patient cells that the drug, a hypoxia-inducible factor prolyl hydroxylase inhibitor, activates HIF and increases mutant *TPI1* mRNA levels as determined by RNAseq [21] supporting the conclusion that this directly explains the elevation in TPI protein and activity. 

The compassionate use of vadadustat was in a single patient; nonetheless, important results were obtained. The patient experienced a slow dose escalation of Vafseo from 75 mg to 600 mg over the course of many months and was carefully monitored, including regular bloodwork. Notably, the patient, who was the first pediatric human and first TPI Df patient to receive the drug, had no adverse events from the treatment. Vafseo has a black box warning that requires careful monitoring of Hb to ensure levels did not escalate. As Vafseo’s approved use is to treat anemia, there was concern that Hb would increase to unsafe levels before a beneficial increase in TPI was observed. For safety, Hb was monitored to ensure it did not exceed the upper normal limit for age/sex of the patient. Surprisingly, Hb did not increase significantly during the course of treatment (Figure 9) and, importantly, treatment was associated with a progressive increase in residual enzymatic activity measured longitudinally, accompanied by parallel neurological improvement without detectable toxicity (Table 1 and Figure 9). Mild persistent elevation of creatine kinase was noted throughout follow-up without evidence of progressive neuromuscular involvement.

As previously published, the patient exhibited neuroimaging abnormalities that indicated selective vulnerability of basal ganglia structures, particularly the putamen, and are highly consistent with the patient’s predominant dystonic phenotype, suggesting tissue-specific neuronal susceptibility associated with this atypical and largely neurological form of TPI deficiency [17]. Although direct quantitative comparison between baseline and follow-up values is limited, the progressive increase observed during serial measurements performed in the same laboratory strongly supports a genuine biological effect of treatment. Although discontinuing treatment to determine whether activity returns to baseline would be informative, this cannot be done in this case for ethical reasons. These findings suggest that selected residual-function *TPI1* variants may represent therapeutically targetable forms of TPI deficiency. Notably, cessation of school attendance was followed by reduced migraine frequency and improved fatigue, suggesting a possible contribution of environmental sensory overload to her neurological symptom burden.

## 5. Conclusions

This study provides the first *in vivo* proof of concept that pharmacological rescue of mutant TPI protein by the clinically approved PHD inhibitor vadadustat (Vafseo) can restore residual TPI enzymatic activity in a patient with TPI deficiency. The concordance between preclinical efficacy in patient-derived cells and an established TPI Df mouse model, biochemical restoration of TPI activity, and clinically meaningful neurological improvement supports further evaluation of PHD inhibitors as potential disease-modifying therapies for TPI deficiency. Treatment with Vafseo was associated with a progressive increase in residual erythrocyte TPI activity, from 42 U/g Hb before treatment initiation to 237.4 U/g Hb at peak response, together with improved motor control, including improved upper-limb dexterity, recovery of functional grasping, complete resolution of myoclonic-like movements, and partial reduction in dystonia. No hematologic, hepatic, renal, or metabolic toxicity, nor treatment-related adverse events, were observed despite dose escalation to 600 mg/day. Although the mechanism underlying the neurological benefit remains uncertain, the improvement in motor control despite preserved muscle strength suggests that additional mechanisms beyond skeletal muscle rescue may contribute to the clinical response. Interpretation of therapeutic efficacy remains limited by the single-patient design and the absence of standardized longitudinal neurological outcome measures. Nevertheless, this work establishes a compelling translational proof of concept and provides a strong rationale for further clinical evaluation of PHD inhibitors as a therapeutic strategy for TPI deficiency.

## Figures and Tables

**Figure 1:**
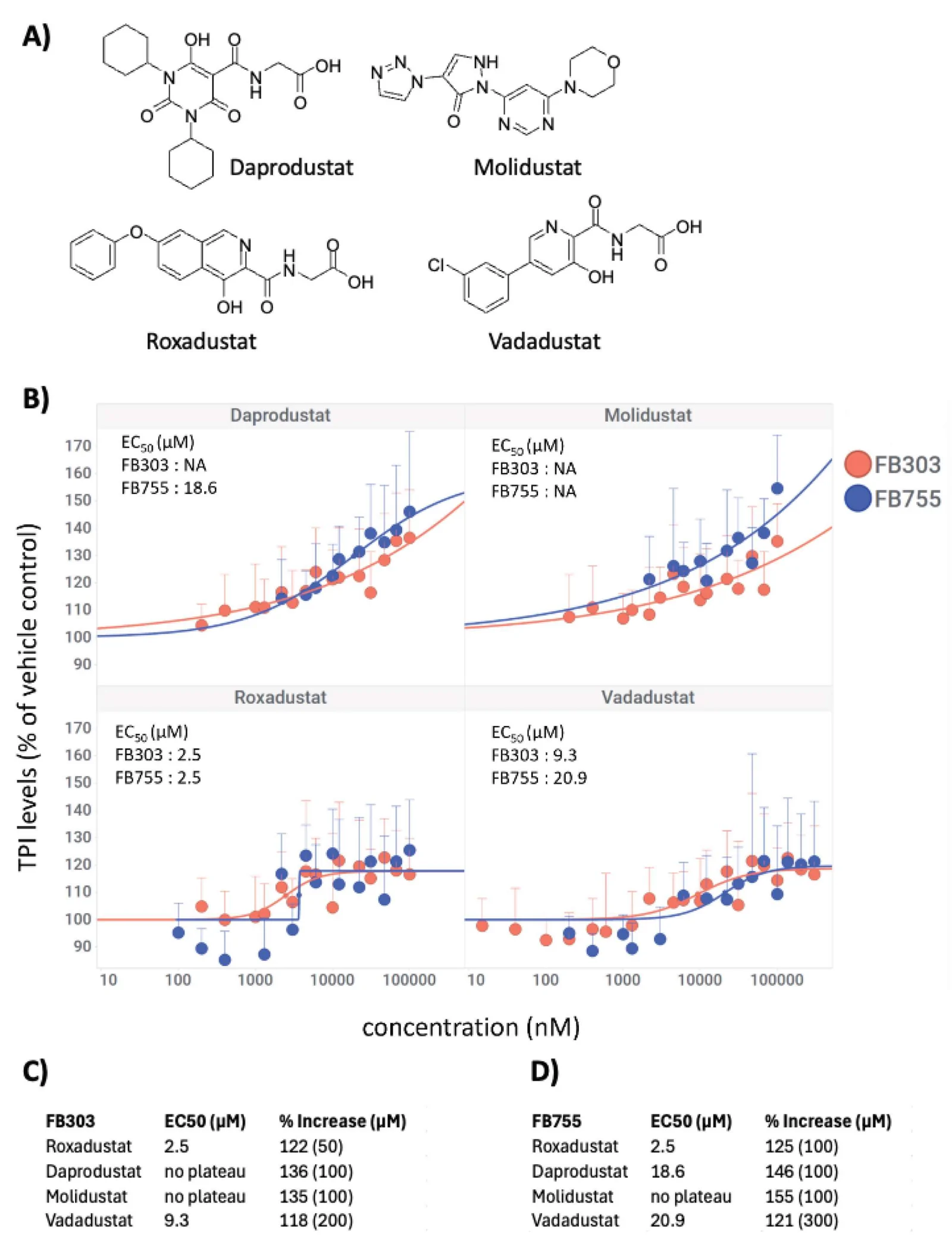
PHDIs increase TPI in patient fibroblasts. (**A**) Structures of daprodustat, molidustat, roxadustat, and vadadustat; the compounds tested in the dose–response curve. (**B**) Ten-point dose responses showing increases in TPI in patient FB303 cells (Red: E105D/E105D) and FB755 cells (Blue: Q181P/E105D) with 4 different PHDIs. All 4 compounds appear active, although there are modest differences in their EC50s and potency in FB303 cells and FB755. The curves were fit and analyzed using Spotfire (version 14.0.2, Revvity, Inc., Waltham, MA USA). In some cases, the response did not plateau, so an EC50 could not be calculated. (**C**) Percent increase in FB303 cells after treatment with four PHDI’s. (**D**) Percent increase in FB755 cells after treatment with four PHDIs.

**Figure 2:**
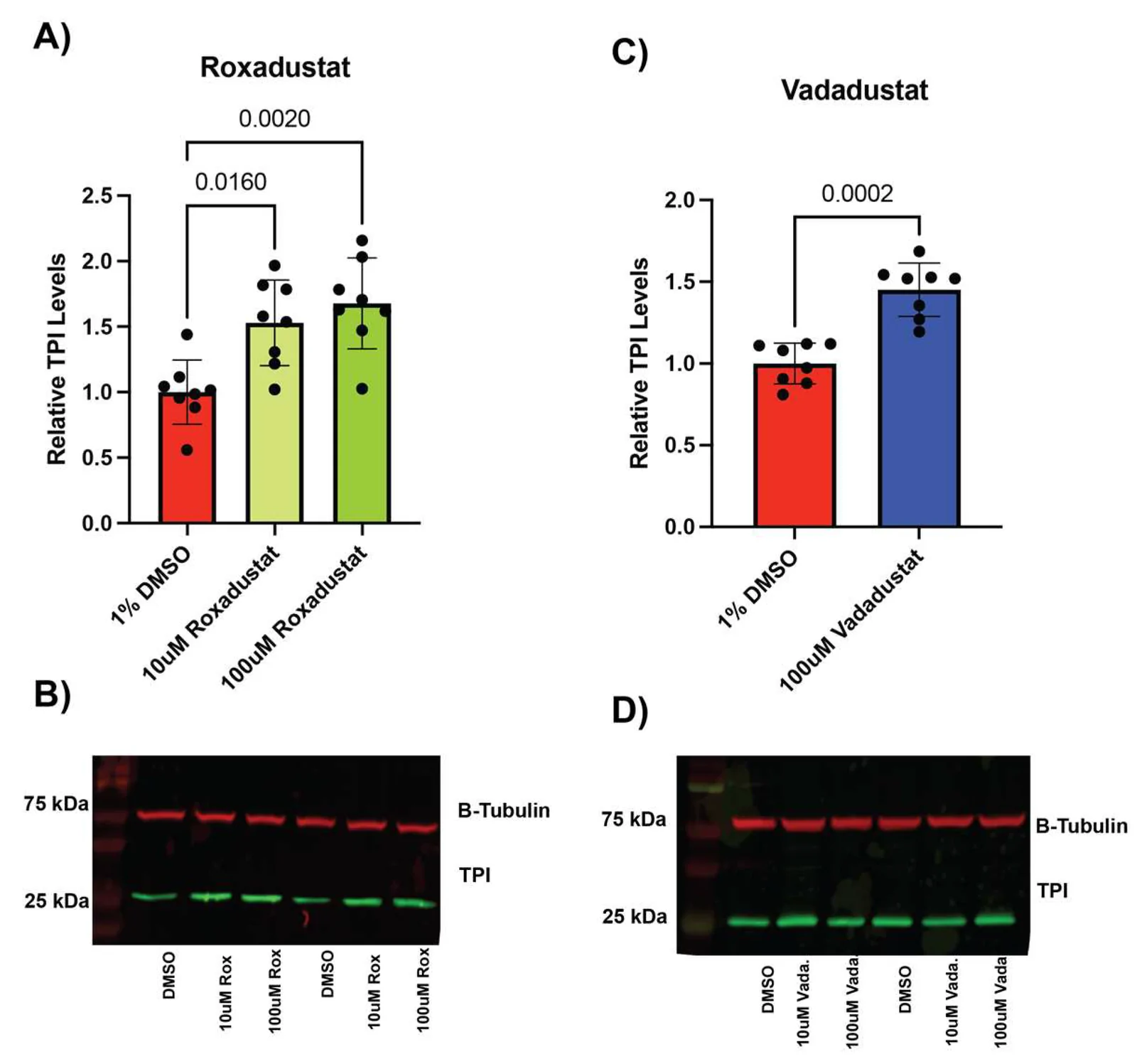
PHD inhibitors roxadustat and vadadustat increase TPI protein levels in human fibroblasts. (**A**) Western blot analysis shows a significant ~53% increase with 10 μM roxadustat treatment, and a ~68% increase with 100 μM roxadustat treatment compared to DMSO vehicle controls. (**B**) Representative blot used in roxadustat treatment analysis. Kruskal–Wallis test was used to determine significance. *p*-values are shown; *n* = 8 for each group; error is Std. Dev. (**C**) Western blot analysis demonstrates a ~45% significant increase in relative TPI protein levels after 100 μM treatment with vadadustat compared to DMSO vehicle controls. (**D**) Representative blot utilized in vadadustat treatment analysis. A two-tailed Mann–Whitney test was used to determine significance. *p*-values are displayed; *n* = 8 for each group; error is Std. Dev.

**Figure 3:**
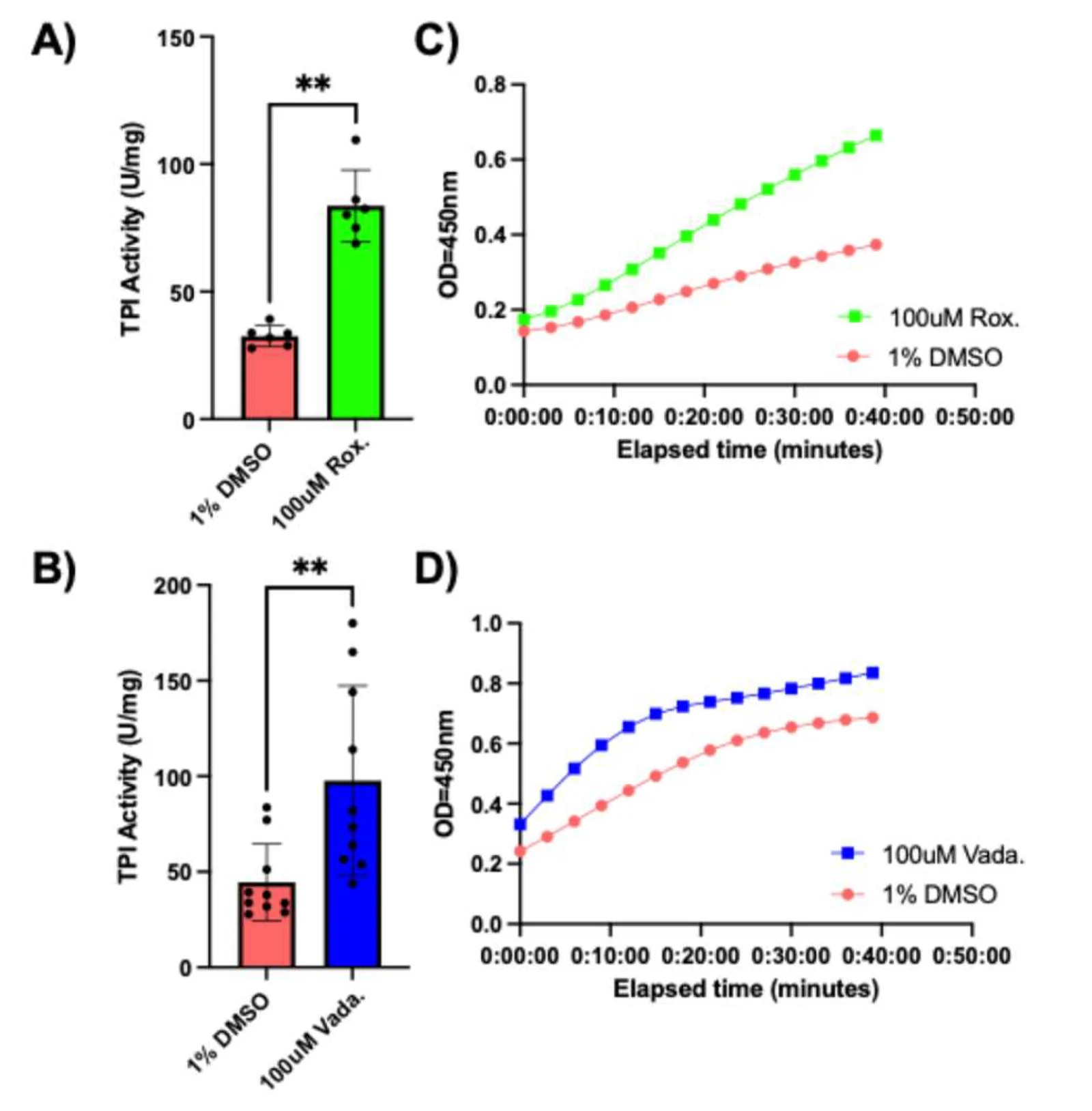
Roxadustat and vadadustat increase TPI activity in patient cells. TPI activity analysis shows a significant increase in protein lysate activity (U/mg) when treated with (**A**) 100 μM roxadustat or with (**B**) 100 μM vadadustat compared to the DMSO vehicle control. Mann–Whitney test was used to determine significance. ** is *p* < 0.01. Error is Std. Dev. (**C**,**D**) Kinetic plots are shown for roxadustat and vadadustat, respectively.

**Figure 4:**
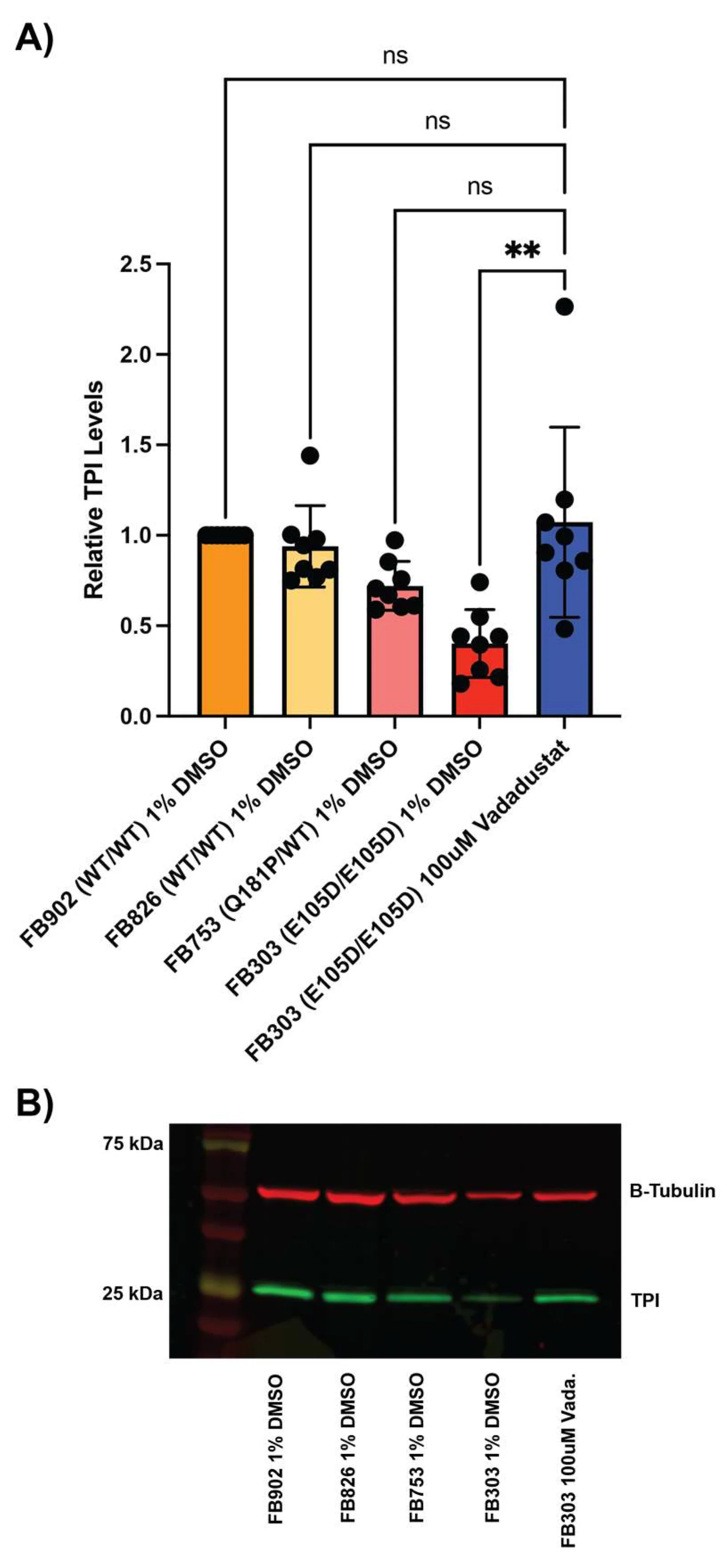
Vadadustat treatment increases FB303 TPI protein to wild-type levels. (**A**) Western blot analysis demonstrates a significant increase in relative TPI protein levels in FB303 patient cells after vadadustat treatment compared to FB902 (WT/WT), FB826 (WT/WT), FB753 (Q181P/WT), and FB303 (E105D/E105D) DMSO vehicle controls. (**B**) Representative blot used in vadadustat treatment analysis comparing treatment levels to wild-type and unaffected heterozygote levels. Kruskal–Wallis test was used to determine significance. ns = not significant, ** = *p*-value less than 0.01, *n* = 8 for each condition, error is Std. Dev.

**Figure 5:**
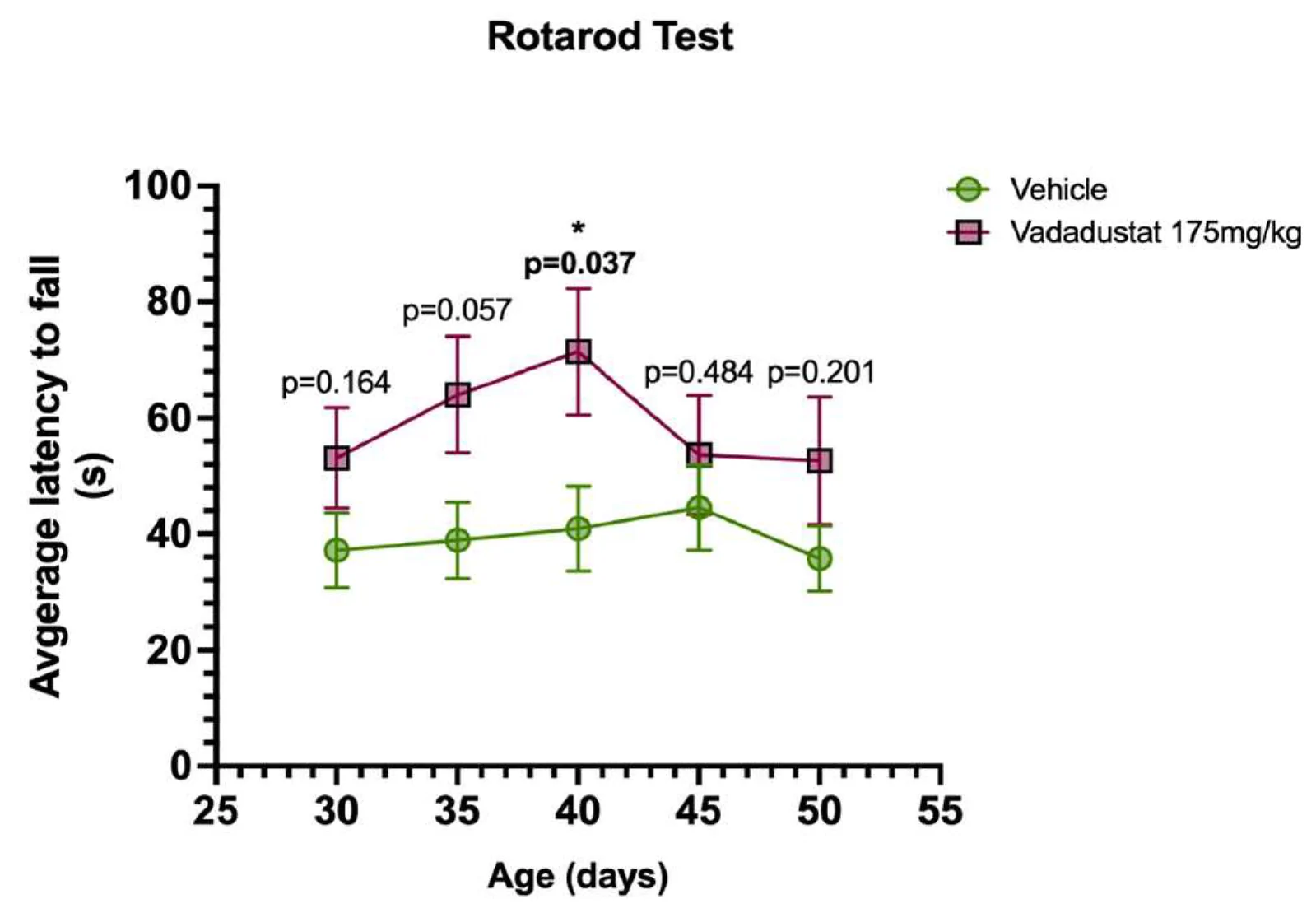
Locomotor function of *TPI1^E105D/E105D^* mice improves with vadadustat. Average latency to fall via rotarod assay in seconds (s) following daily treatment with DMSO vehicle control or vadadustat from 30 to 50 days of age. *n* =  8 animals per group with equal numbers of males and females; data was pooled due to the absence of sex differences. Data were analyzed via a two-way repeated-measures ANOVA: treatment *p* = 0.096, time *p* = 0.259, interaction *p* = 0.410. * is *p* < 0.05.

**Figure 6:**
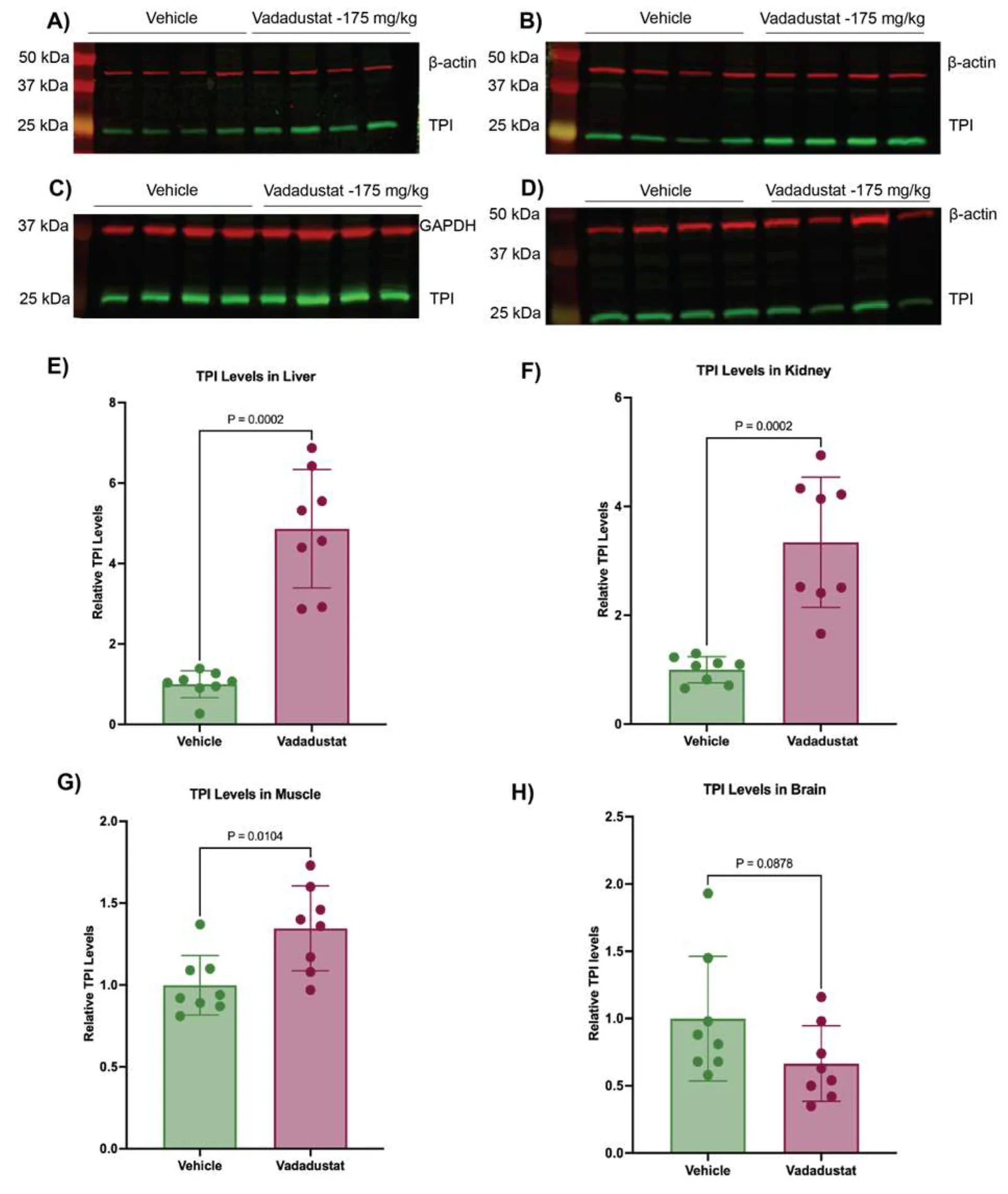
Western blot analysis of TPI levels in *Tpi1^E105D/E105D^* mice treated with vadadustat (175 mg/kg) or the DMSO vehicle control. Panels (**A**–**D**) show representative western blots of liver, kidney, skeletal muscle, and brain tissue, respectively. *n* = 8 animals per group. Panels (**E**–**H**) are quantifications of TPI levels from Western blot analyses.

**Figure 7:**
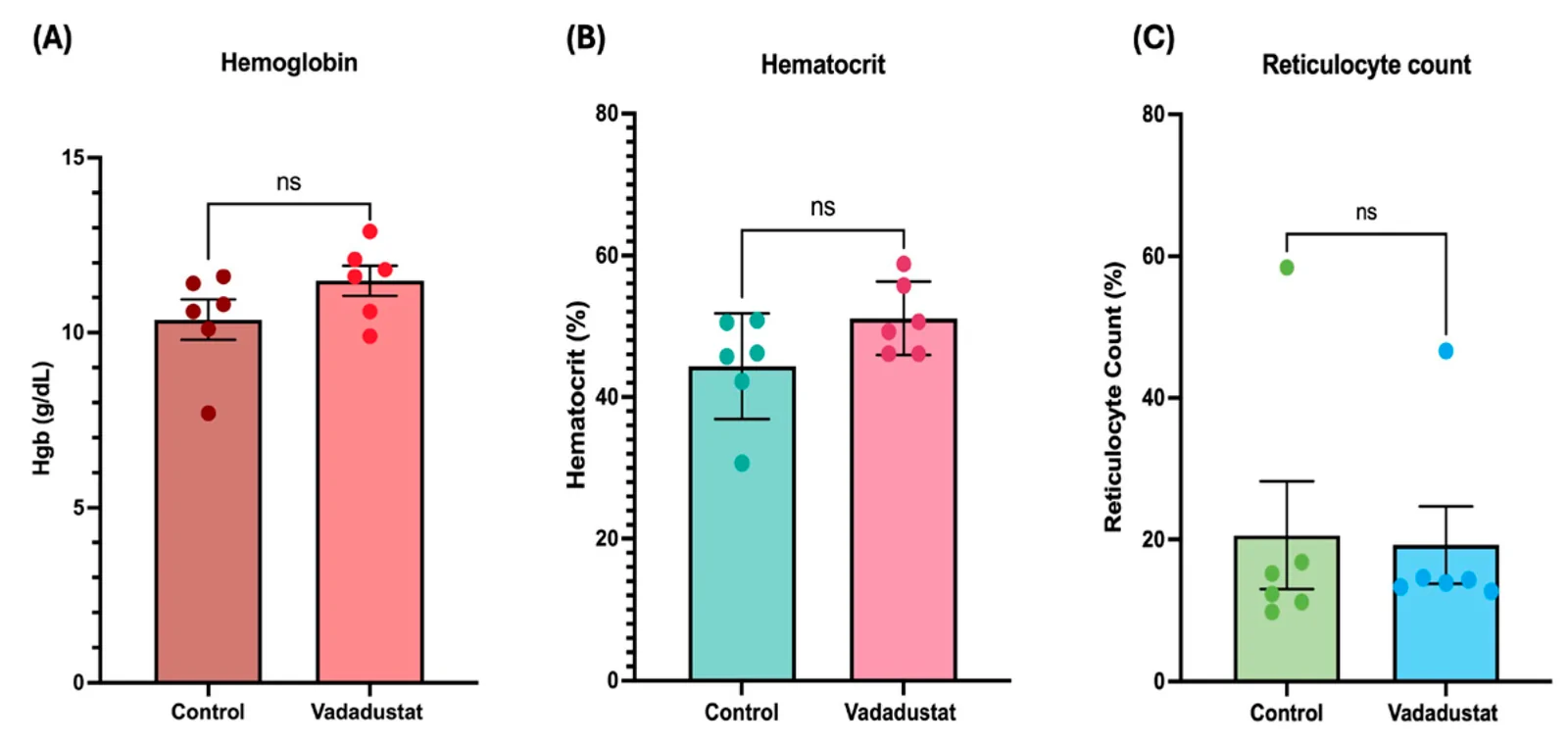
Hematologic analyses for *Tpi1^E105D/E105D^* mice treated with vadadustat (175 mg/kg) or DMSO vehicle control**.** Panels (**A**–**C**) report results for hemoglobin, hematocrit, and reticulocyte count, respectively, with daily vadadustat or DMSO vehicle control administration. *n* = 6 animals per group. Mann–Whitney test was used to determine significance. ns is not significant.

**Figure 8:**
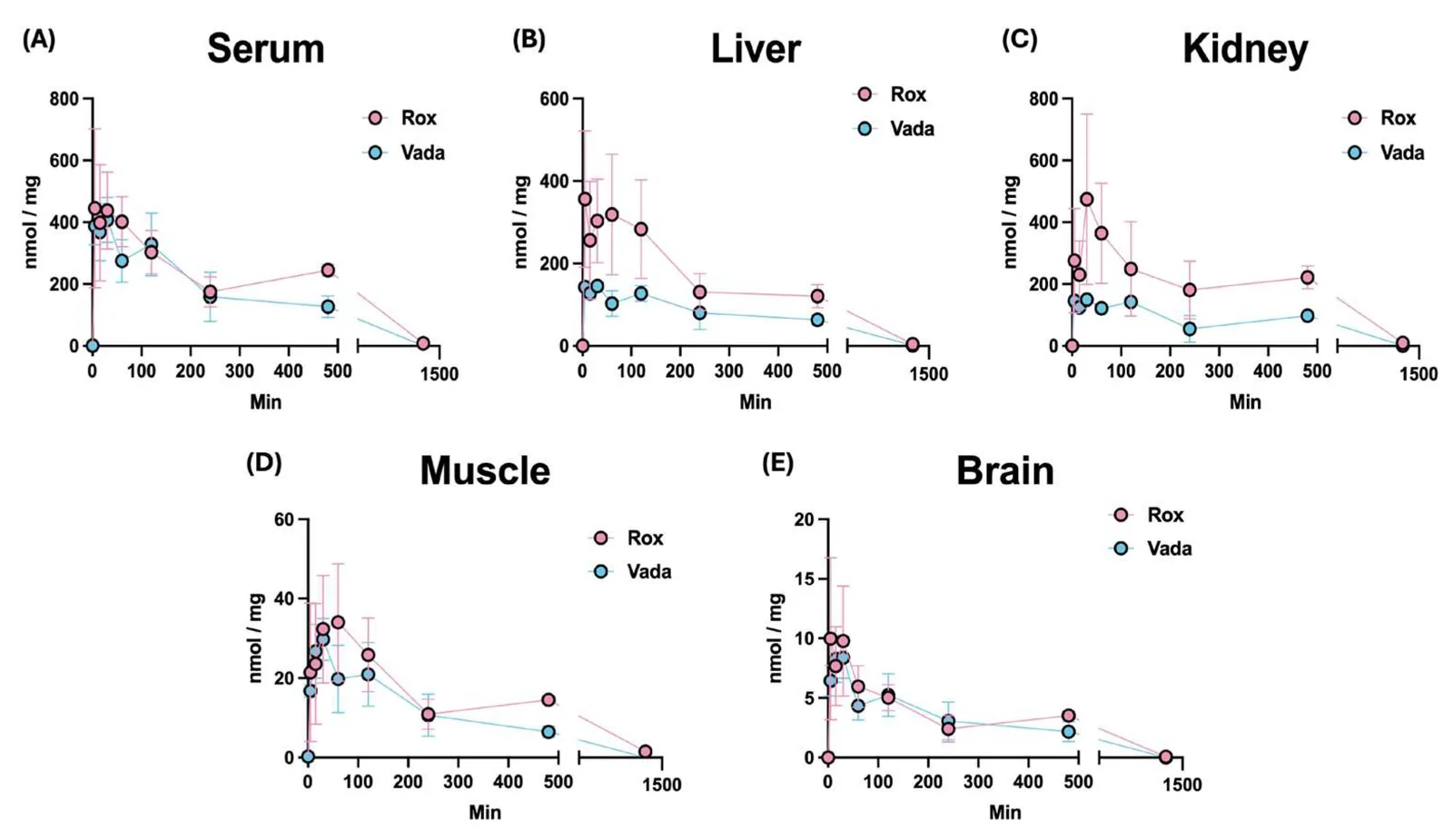
Tissue distribution analyses of PHD inhibitors. Pharmacokinetics of roxadustat and vadadustat were examined at several time points: 0.08, 0.25, 0.5, 1, 2, 4, 8, and 24 h. Panel (**A**) represents serum levels, while panels (**B**–**E**) show tissue distribution across tissues. Error shown is SEM.

**Figure 9:**
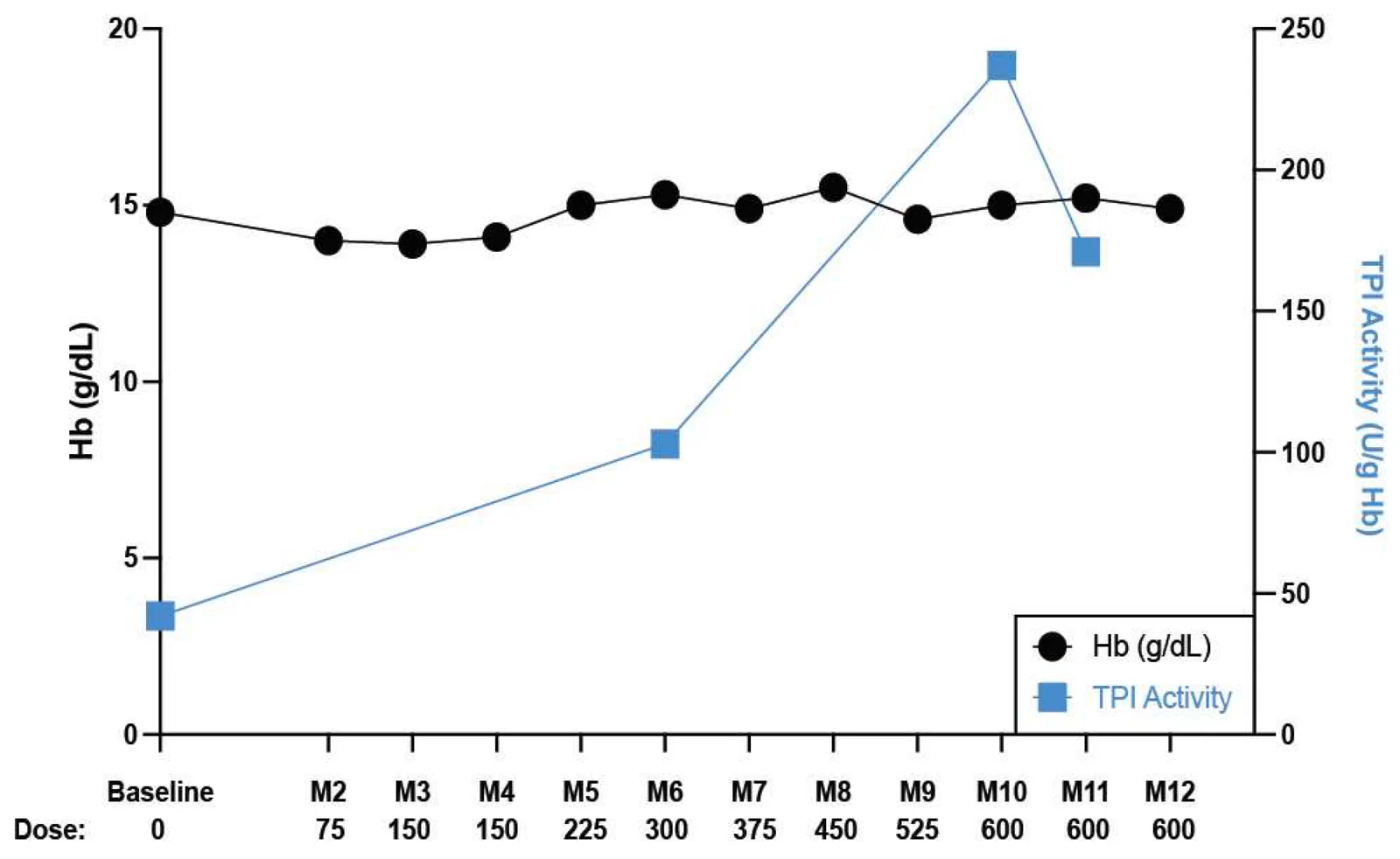
Biochemical effect of Vafseo treatment in compassionate use over the 12 months (M) of monitoring. Hb showed little variability and did not change significantly throughout the treatment (Black). TPI activity showed an increasing trend with a sharp escalation at the 600 mg/day dose. From M10–M12, the patient reported clinical improvement. Note: In M6 (300 mg/day), the patient did not exhibit clinical improvement, suggesting that significantly more than a doubling of TPI activity was needed for a noticeable benefit or that sustained elevation was required. Dose is the amount of Vafseo administered one time in mg/day.

**Table 1:** Mass spectrometry parameters for the quantification of roxadustat and vadadustat. Q1, parent ion; Q3, fragment ion; DP, declustering potential; CE, collision energy.

Q1	Q3	Time (ms)	Analyte	DP	CE
353	307	60	Roxadustat 1	110	24
353	278	60	Roxadustat 2	115	25
353	250	60	Roxadustat 3	113	36
307	261	60	Vadadustat 1	65	22
307	232	60	Vadadustat 2	72	25
307	204	60	Vadadustat 3	72	33
358	312	60	Roxadustat-d5 1	110	24
358	283	60	Roxadustat-d5 2	115	25
358	255	60	Roxadustat-d5 3	113	36
310	264	60	Vadadustat 13C2 15N 1	65	22
310	235	60	Vadadustat 13C2 15N 2	72	25
310	207	60	Vadadustat 13C2 15N 3	72	33

**Table 2:** Serial clinical laboratory monitoring during progressive Vafseo dose escalation.

Parameter (Normal Range)	T0	M2	M3	M4	M5	M6	M7	M8	M9	M10	M11	M12	M13
Vadadustat (mg/day)	0	75	150	150	225	300	375	450	525	600	600	600	600
WBC (4.5–11.4 × 10^9^/L)	7.54	6.45	6.28	6.19	5.95	6.03	6.25	6.71	7.34	6.02	6.38	6.89	7.22
Hb (12.0–15.4 g/dL)	14.8	14.0	13.9	14.1	15.0	15.3	14.9	14.5	14.6	15.0	15.2	14.9	15.3
Hct (35.5–45.0%)	44.1	40.7	40.8	41.2	42.9	44.1	41.5	39.9	40.9	43.4	43.6	42.7	44.5
PLT (160–385 × 10^9^/L)	263	240	241	259	244	241	247	383	257	243	231	238	265
ANC (1.7–7.9 × 10^9^/L)	4.61	2.03	2.17	2.38	2.35	2.47	2.48	2.61	2.69	2.73	2.76	2.85	3.20
CK (<123 U/L)	102	----	143	138	143	153	227	296	141	182	171	164	193

Abbreviations: WBC, white blood cells; Hb, hemoglobin; Hct, hematocrit; PLT, platelets; ANC, absolute neutrophil count; CK, creatine kinase.

## Data Availability

All data being reported are complete data sets. Additional information and protocols are available upon request. Redacted clinical data are available on request from the corresponding author due to ethical/privacy concerns.

## Abbreviations

The following abbreviations are used in this manuscript:

TPI	Triosephosphate Isomerase
TPI Df	Triosephosphate Isomerase Deficiency
Hb	Hemoglobin
PHD	Prolyl hydroxylase domain
HIF	Hypoxia-inducible factor
CKD	Chronic kidney disease
G3P	glyceraldehyde-3-phosphate
DHAP	dihydroxyacetone phosphate
